# Video exposure through virtual reality can improve older people’s ability to manage postural instability caused by distortive visual environments

**DOI:** 10.1371/journal.pone.0306834

**Published:** 2024-08-21

**Authors:** Jenny Älmqvist Nae, Anastasia Nyström, Francesca Luccini, Måns Magnusson, Eva Ekvall Hansson

**Affiliations:** 1 Department of Health Sciences, Lund University, Lund, Sweden; 2 Department of Otorhinolaryngology Head and Neck Surgery, Skåne University Hospital, Lund, Sweden; 3 Department of Behavioral Sciences and Learning, Linköping University, Linköping, Sweden; 4 Department of Clinical Sciences Lund, Lund University, Lund, Sweden; UFPE: Universidade Federal de Pernambuco, BRAZIL

## Abstract

In older adults, age-related degenerative processes and disorders often degrade some sensory systems more than others, which can make postural control disproportionally dependent on one kind of sensory information. The study aims were to investigate 1) the postural stability when healthy older adults were repeatedly exposed to a video in an immersive virtual reality (VR) environment, and 2) the relationship between stability during VR video exposure and self-reported physical activity, balance confidence, and nausea during VR. Twenty-seven older adults (18 females, mean age 71.3 years (SD 4.4)) watched a 120-second VR video 5 times with 10 minutes between sessions, while standing on a force platform recording their stability. The first VR video session produced a marked stability challenge, reflected by significantly increased use of anteroposterior and lateral total (p<0.001) and high frequency (p<0.001) energy compared with the control test quiet stance eyes open. However, repeated VR video sessions produced a multidimensional decrease in used total (p<0.001), low (p = 0.002), and high frequency energy (p<0.001). Participants used more energy in anteroposterior compared with lateral direction across sessions within all spectral ranges (p<0.001). Participants with higher physical activity level used less low frequency energy in anteroposterior direction during VR video session 1 (p = 0.033). No association was seen between balance confidence or nausea during VR and energy used during VR video sessions 1 and 5. Healthy older adults adapt fast to distortive visual environments, and thus, CNS can utilize the information provided by a few repeated VR video sessions into suitable movement strategies that have a simultaneous multidimensionally positive effect. VR may introduce numerous opportunities to customize novel rehabilitation approaches to address when the visual system causes and/or suffers from issues. However, a common problem for the older adult was that about 33% of the participants became nauseated by the VR video stimuli.

## Introduction

Postural control is defined as “the act of maintaining, achieving, or restoring a state of balance during any posture or activity”. Postural stability is maintained by utilizing the appropriate sensorimotor strategy to stabilize the center of mass so it addresses each individual challenge of balance [1]. The central nervous system (CNS) has an important role in stabilizing the body when moving and interacting with the environment [1]. To maintain balance (i.e., postural stability), the CNS must detect and stabilize body movements by means of information produced by cues from various physiological sensory systems, e.g., the vestibular, somatosensory, and visual systems [1–3]. These systems provide the CNS with information about 1) the position and movement of the head (vestibular system) [2], 2) body positions and movements through receptors in for example muscles, tendons, joints and the skin (somatosensory system) [4], and 3) the spatial location of the body in the environment as well as the relative movement and positions of the body in this environment (visual system) [3]. Among these 3 systems, the visual system is suggested to have an essential role in maintaining postural stability during a quite stand test [5]. The CNS is processing this information from the sensory systems concurrently. Hence, it is prepared to address any detected imbalances and prevent potential falls [1, 2].

Falls are one of the leading causes of unintentional injury and death in older adults [6]. Older adults (65+) are more inclined to be injured by a fall [7]. Approximately 30–40% of people over 65 years of age report that they have suffered falls in the past year [8]. A large proportion of these falls (in Sweden 40%) occur at home [7]. The risk of falling increases if sensory and motor systems, essential for a person’s ability to stand and maintain balance, are affected by illness or disease and by age-related degenerative processes and disorders [1]. Ageing is associated with functional distortions which often simultaneously affect several sensory and motor systems (e.g., vestibular, somatosensorial, and visual systems, muscle activation and force output). This results in an impaired postural stability due to difficulties in integrating the partially flawed information received by the sensory systems and from the reduced ability to produce the muscle output or stabilizing movement patterns required for maintaining stability [9–11].

Systematic reviews and meta-analyses on the effect of balance training in older adults have shown that sensorimotor training that instigates reweighting to the most reliable sensory information sources is beneficial for improving static and dynamic postural stability during standing and walking [12]. Other successful concepts involve making postural control more proactive (i.e., to make actions in anticipation of a predicted perturbation, e.g., stepping over an object), and making postural control better at selecting and controlling reactive responses (i.e., to make a suitable response to an unpredicted disturbance, e.g., slipping or tripping) [12]. Training utilizing virtual reality (VR) has recently been introduced as a novel approach to enhance postural stability and instigate sensory reweighting [13]. Repeated exposure to distorted visual information can be used to make CNS adjust the processing and integration of sensory information sources, and thus, reduce the postural instability caused by poor ability to handle distortive visual information [13]. When the concept of using VR as a tool for balance training and rehabilitation of older adults was recently investigated in a systematic review and in a randomized controlled trial, the authors reported encouraging results [14, 15]. When older adults with balance disorders and high risk of falling performed training utilizing VR for two months, the training resulted in improvements in functional balance, anterior reach, mobility, as well as reduced dizziness [14]. The review paper reported that functional balance in general can be trained and evaluated using VR systems [15]. However, the review paper also reported disparities between the training results achieved for healthy older adults and for older adults with balance impairments [15]. Hence, it was suggested that more research should be performed to develop efficient training protocols and guidelines for how to use VR for balance assessments and for training older adults [15].

A recent study on healthy young adults used a protocol with a repeated exposure (five VR sessions) to an immersive VR video which resulted in a fast adaptation in postural stability [16]. The authors reported a significant reduction in both lateral and anteroposterior energy used during the fifth VR session compared with the first VR session. The results suggest that the CNS can adapt to VR stimuli and at the same time adapt the stability multidimensionally [16]. However, whether the adaptation of postural stability occurs as fast and multidimensionally in healthy older adults when exposed to immersive VR stimulus, by using the same protocol, has not been investigated. Moreover, it is largely unknown whether personal characteristics, e.g., physical status and susceptibility to migraine and motion sickness, affect the subject’s ability to address distortive visual environments [16]. Thus, the aims of this study were to 1) investigate the postural stability when healthy older adults were repeatedly exposed to a video in an immersive virtual environment, and 2) determine the relationship between the stability during VR exposure and self-reported physical activity, balance confidence, and nausea during VR.

## Materials and methods

### Ethical consideration

The study was performed in accordance with the latest Declaration of Helsinki and received ethical approval from the Ethical Review Authority in Sweden (Dnr: 2018/320). All participants gave their written informed consent prior to participation.

### Study design

This experimental study used a repeated measures design was used to evaluate the participants’ adaptation capacity to the VR video stimulus, and the influence of repetition and direction on postural stability.

### Participants

Participants were included if they were older adults (≥65 years), considered themselves healthy, and had no prior history of diseases that could affect their balance, e.g., a known history of vertigo or neurological diseases. The participants were screened for health disorders by using a custom-made questionnaire. Participant recruitment was performed between 1^st^ of February to 16^th^ of December 2022 using advertisements on Facebook, and through contacts with friends and family.

One exclusion criterion was if the participant suffered from a peripheral vestibular dysfunction. This was assessed by the video head impulse test (vHIT), performed by an experienced assessor with more than 5 years of clinical experience in performing vHIT tests [17, 18]. The vHIT test is a validated reliable method used for analyzing the functionality of the semicircular canals, where each semicircular canal (lateral, anterior, and posterior) is analyzed separately [17, 18]. During this test, the examiner rotates the participant’s head in short, fast (>150 deg/second) thrusts, while the participant fixates on a static visual target. The head thrusts are performed in unpredictable directions in the plane of the semicircular canals to be investigated. To enable the participant to maintain fixation on the target, a very fast vestibulo-ocular reflex (VOR) detects the head thrusts and moves the eyes in the opposite direction with the same velocity. The eye movements were recorded with a high-speed camera, and the head movements were recorded with a gyro sensor [18]. Participants were excluded from the study if the recorded gain was below 0.7 in any semicircular canal direction, as this was regarded as a sign of pathological vestibular dysfunction. The vHIT test was not performed on participants who reported that they occasionally had neck stiffness.

### Procedure

The participants were referred to the otorhinolaryngology laboratory at Lund University Hospital for the testing. The testing required approximately 90 minutes per person, and all the tests were performed on the same day in the order described below. As part of a concept evaluation, the participants answered questions regarding age, sex, weight, height, prevalence of migraine and motion sickness, prior VR experience, if nauseated by the VR when exposed to it, and physical status as described by the Activity-specific Balance Confident scale (ABC) and Frändin/Grimby questionnaire scores.

#### Balance confidence

The participants were asked to complete the ABC scale to evaluate their confidence in completing tasks without losing their balance. The ABC scale is a self-reported questionnaire with 16 items, and is validated for use in older adults to assess their level of functioning [19]. The ABC scale has shown good internal consistency, test-retest reliability, and convergent and criterion validity [19]. A high level of functioning is defined as scoring >80% on the ABC scale [20].

#### Physical activity level

The Frändin/Grimby physical activity scale is a self-reported questionnaire aimed to be used in older adults and people with reduced physical functioning to measure the physical activity level, taking into consideration both intensity and duration of the physical activity [21]. The scale is divided into 6 levels; 1) Hardly any physical activity; 2) Mostly sitting, sometimes light activities, e.g., household activities, a walk or light gardening; 3) Light physical exercise around 2–4 h a week, e.g., walks, ordinary gardening including walks to and from shops; 4) Moderate exercise 1–2 h a week, e.g., jogging, swimming, heavy gardening, or light physical activities more than 4 h a week; 5) Moderate exercise at least 3 h a week, e.g., tennis, swimming, jogging; and 6) Hard or very hard regular exercise several times a week, where the physical exertion is great, e.g., jogging [21, 22]. The scale has been validated to perform valid assessments of physical performance with good test-retest reliability [21, 22].

#### Posturography assessment and virtual reality procedure

**Fig 1:**
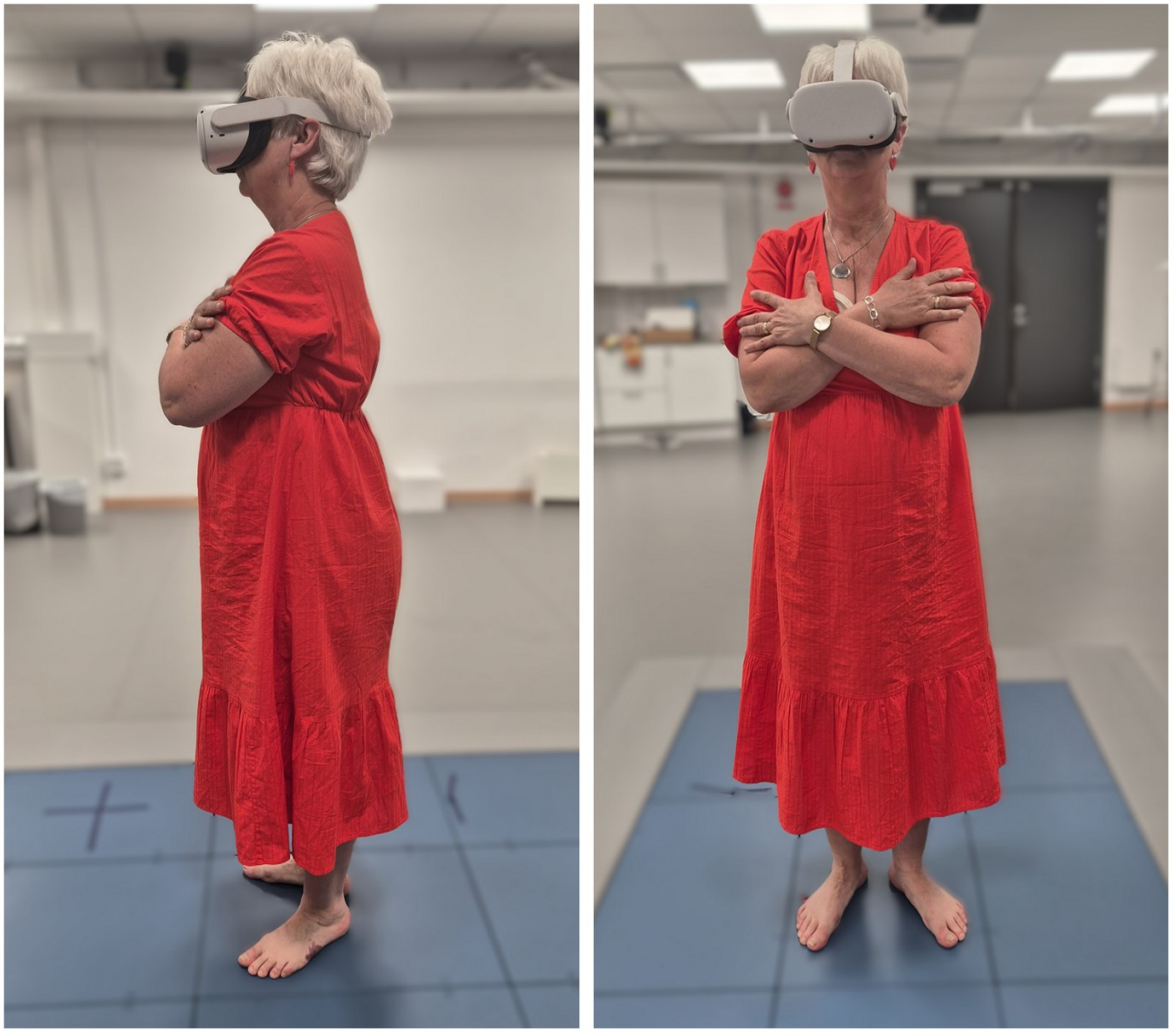
Standardization of the posturography assessment and virtual reality procedure. The participants were standing in a relaxed position, with their arms folded over their chest, and they were instructed to keep their head straight forward while watching the VR video. The force platform recorded the participants’ postural stability and the body movements to maintain balance while watching the VR video.

The Oculus Quest 2 VR headset (Facebook Technologies, LLC, Menlo Park, California, USA) used in the study tracks the head movements in 360-degree range, and thus, any change in head direction produced an identical direction change in the VR movie displayed to the subject. First, the participants were shown a winter landscape 360° video while sitting. This was done to calibrate the sharpness of the images displayed through the VR visor before performing the posturography assessment. If participants were using glasses, they were given the choice to keep or remove the glasses. All participants choose to remove them due to limited space inside the VR glasses. A question regarding whether the participants could see the video was asked to ensure that they could see the video clearly without glasses. The participants were then instructed to stand on a force platform without shoes and with their feet positioned with a slight outward rotation (approximately 30 degrees) using guidelines on the force platform, and thus, to adhere to a standardized posturography test protocol [16, 23, 24]. The force platform (AMTI HPS 464508, AMTI Europe GmbH, Helmstadt-Bargen, Germany) recorded the participants’ postural stability and the body movements to maintain balance. The participants were standing in a relaxed position, with their arms folded over their chest, and they were instructed to keep their head straight forward while watching the VR video (Fig 1). Performing substantial head movements in a VR environment may produce additional strain for the participants. Thus, to make the test conditions standardized and as similar as possible for all subjects, the participants were instructed to look straight ahead during the VR video sessions.

The participants thereafter performed seven posturography tests on the force platform. Five assessments were made while exposed to the VR video stimuli, repeatedly watching a 360° video of “walking in the grocery store”, where the walk included numerous stops/starts and quick turns to the right and left around high shelves. The same video was shown for 120 seconds each session with a 10-minute rest period between sessions. The participants were allowed to sit down and rest between repeated VR video sessions. Two control tests (quiet stance with eyes open, quiet stance with eyes closed without VR headset), were performed in a randomized order after the five VR video sessions were performed. During the control tests, the participants were instructed to stand in the same way as during the VR video sessions, once with their eyes closed and once focusing on a target positioned at eye level on the wall in front of them. The control tests were used for assessing the normal postural sway and to provide a relative reference for the stability recorded when the participants were exposed to distortive visual information from the VR video.

### Analysis

The stability and the adaptation in anteroposterior and lateral directions during repeated VR video stimuli were determined by analyzing the variance of anteroposterior and lateral torque values. The torque variance values reflect the energy used towards the force platform to maintain stability [25, 26]. In addition, spectral separations were performed to describe the smooth corrective changes of posture (i.e. low frequency energy <0.1 Hz) and the fast corrective movements to maintain balance (i.e. high frequency energy >0.1 Hz) [27], using a fifth-order digital Finite duration Impulse Response (FIR) filter. The torque variance values were normalized to height and weight by using the subjects’ squared height and squared weight, as height and weight influence body sway [16, 25, 28]. The squared components in the variance formula made it necessary to normalize with squared parameters to achieve unit agreement.

### Statistical analysis

The stability in anteroposterior and lateral directions during the control tests and the five VR video sessions were analyzed using repeated measures GLM ANOVA on log-transformed normalized torque variance values, reflecting the total, low frequency, and high frequency energy, respectively. The analysis method was used after ensuring that all dataset combinations analyzed in the study with this statistical method produced model residuals that had normal or close to normal distribution, thus validating whether the GLM ANOVA method was appropriate for analyzing the data [29]. The main factors and factor interactions analyzed for VR sessions were: ‘Repetition’ (Session 1…5; degree of freedom (d.f.) 4); and ‘Direction’ (Anteroposterior vs. Lateral; d.f. 1). In the analyses, the log-transformed normalized total, low frequency, and high frequency torque variance values were dependent variables, and the model parameter Repetition and Direction were within-subjects independent variables. For the control tests, the main factors and factor interactions analyzed were: ‘Vision’ (Eyes closed vs. Eyes open; d.f. 1); and ‘Direction’ (Anteroposterior vs. Lateral; d.f. 1). In the analyses, the log-transformed normalized total, low frequency, and high frequency torque variance values were dependent variables, and the model parameter Vision and Direction were within-subjects independent variables.

The Wilcoxon matched-pairs signed-rank test (Exact sig. 2-tailed) was used for post hoc within-subjects analyses of the accumulated effects of repeatedly performing VR video Sessions i.e., analyzing the adaptive changes between VR video Session 1 and VR video Session 5; for determining the difference between VR video Session 1 and the quiet stance Eyes open test; for determining the difference between VR video Session 5 and the quiet stance Eyes open test; and for determining the role of vision and direction [30].

The association between balance confidence (ABC scale) and physical activity level (Frändin/Grimby questionnaire scores) and the stability recorded during VR video session 1 and VR video session 5, respectively, was analyzed with Spearman rank correlation coefficient tests. The Mann-Whitney U (Exact sig. 2-tailed) test was used to evaluate whether nausea during VR video sessions affected the stability recorded during VR video session 1 and VR video session 5, respectively.

In the GLM ANOVA analyses, p-values p<0.05 were considered significant. In the post-hoc analyses, p-values < 0.025 were considered significant after Bonferroni correction. Non-parametric statistical methods were used in the post hoc tests as the Shapiro-Wilk test revealed that some datasets were not normally distributed, and that normal distribution could not be obtained by log-transformation [29].

Sample size analyses of the posturography parameters revealed an effect size of 0.95 which shows that with the p-value set to 0.05 (2-tailed), our study requires 11 subjects to reach a power value of 0.8 for this parameter. The statistical analyses were performed using SPSS (Version 28, IBM Corp, Armonk, NY, USA) and the power analysis was performed with GPower 3.1.9.7.

## Results

Thirty subjects were initially recruited to participate in the study, whereof 27 were included in the final material (9 males, 18 females, mean age 71.3 years (standard deviation (SD) 4.4 years). The participants’ characteristics (age, sex, BMI, prevalence of migraine and motion sickness, VR experience, became nauseated by the VR, the Activity-specific Balance Confident scale (ABC) and the Frändin/Grimby questionnaire scores) are presented in Table 1. Of the thirty participants recruited, three were excluded. One subject was excluded due to revealed pathological vestibular dysfunction. One subject got nauseated by the VR movie during the third test session to the level that the subject had to abort participation, and one subject found, during the first session, the VR movie so immersive that the subject aborted participation.

**Table 1:** Descriptive characteristics, n = 27.

Characteristics	
Age (years), mean (SD)	71.3 (4.4)
BMI, mean (SD)	24.6 (3.3)
Women, n (%)	18 (66.7)
Migraines, n (%) ^a^	7 (26)
Motion sickness, n (%) ^a^	10 (37)
VR experience, n (%) ^a^	6 (22)
Nauseated by VR, n (%) ^a^, ^b^	9 (33)
ABC, mean (SD) ^c^	93.9 (5.7)
Frändin/Grimby, mean (SD) ^a^, ^d^	4.1 (1.0)

^a^ Rated as [No Yes]. The nominal parameter values are presented as: number of subjects (percent of investigated group).

^b^ If answered with Yes, the subject felt nauseated during one or more of the VR sessions.

^c^ The Activity-specific Balance Confident scale (ABC) describes confidence experienced when performing daily tasks, as rated between 0 and 100 percent in steps of 10%.

^d^ The Frändin/Grimby rating scale describes how participants subjectively rate their physical activity from 1 (hardly any physical activity) to 6 (Hard exercise several times a week).

The prevalence of migraine and motion sickness was reported by 26% and 37% of the participants, respectively. Twenty-two percent had prior VR experience and 33% of the participants were nauseated during at least one of the VR video sessions, see Table 1.

### Influence of Repetition and Direction during VR sessions

The repeated measures GLM ANOVA analyses revealed that when the participants repeatedly watched the same VR movie, the energy used within all spectral ranges during the VR sessions gradually decreased significantly; 49% gradual decrease in total energy (p<0.001); 10% decrease in low frequency energy (p = 0.002) and 64% decrease in high frequency energy (p<0.001) (Table 2). The participants used significantly more energy in anteroposterior direction than in lateral direction within all spectral ranges during the VR video sessions; 85% more total energy (p<0.001), 168% more low frequency energy (p<0.001) and 45% more high frequency energy (p<0.001). The interaction between the main factors Repetition x Direction showed that the energy used during the VR video sessions decreased more in lateral direction (decreased by 66%) than in anteroposterior direction (decreased by 31%) in total energy (p = 0.041) and decreased more in lateral direction (decreased by 79%) than in anteroposterior direction (decreased by 50%) in high frequency energy (p<0.001).

**Table 2:** Influence of Repetition and Direction during VR video sessions.

VR stability ^a^	Repetition ^b^	Direction ^b^	Repetition x Direction ^b^
Total	**< 0.001 [22.6]**	**< 0.001 [99.1]**	**0.041 [4.7]**
Low <0.1 Hz	**0.002 [12.5]**	**< 0.001 [99.6]**	0.092 [3.1]
High >0.1 Hz	**< 0.001 [43.5]**	**< 0.001 [57.0]**	**< 0.001 [28.9]**

VR = virtual reality

^a^ Repeated measures GLM ANOVA analyses of how the stability was affected by main factors “Repetition” and “Direction” alone and by their main factor interactions during the VR video sessions.

^b^ Presented as: p-values and [F-values]. Found significant effects are marked in bold text.

### Post hoc evaluation of the initial effects and of adaptation to VR videos

Post-hoc analyses performed to determine the initial effects of virtual reality exposure, revealed that the participants significantly increased the energy used during VR video session 1 vs. the quiet stance control test with eyes open, in both anteroposterior and lateral directions. In anteroposterior direction, the total energy was increased by 150% (p<0.001), and the high frequency energy was increased by 540% (p<0.001) during VR video session 1 compared with eyes open (Table 3). In lateral direction, the total energy was increased by 612% (p<0.001), the low frequency energy was increased by 97% (p = 0.010) and the high frequency energy was increased by 2034% (p<0.001) during VR video session 1 compared with eyes open (Table 3).

**Table 3:** Influence of initial VR video and effects of adaptation.

Stability changes	p-value ^a^	VR 1/EO ^b^^,^ ^c^	p-value ^a^	VR 5/VR 1 ^b^^,^ ^d^	p-value ^a^	VR 5/EO ^b^^,^ ^e^
Anteroposterior						
Total	**< 0.001**	**2.50 (0.53)**	**< 0.001**	**0.69 (0.09)**	**< 0.001**	**1.72 (0.31)**
Low <0.1 Hz	0.201	1.36 (058)	0.869	0.92 (0.36)	0.162	1.26 (0.38)
High >0.1 Hz	**< 0.001**	**6.40 (0.82)**	**< 0.001**	**0.50 (0.05)**	**< 0.001**	**3.22 (0.33)**
Lateral						
Total	**< 0.001**	**7.12 (1.72)**	**< 0.001**	**0.34 (0.16)**	**< 0.001**	**2.42 (0.47)**
Low <0.1 Hz	**0.010**	**1.97 (0.44)**	0.746	0.87 (0.30)	0.033	1.71 (0.51)
High >0.1 Hz	**< 0.001**	**21.34 (10.80)**	**< 0.001**	**0.21 (0.06)**	**< 0.001**	**4.48 (2.33)**

^a^ Found significant effects are marked in bold text.

^b^ The quotient values are presented as: group mean quotient (individual quotient SEM shown in brackets).

^c^ The quotient values between VR video session 1/Quiet stance control test with eyes open.

^d^ The quotient values between VR video session 5/VR video session 1.

^e^ The quotient values between VR video session 5/Quiet stance control test with eyes open.

VR = virtual reality, EO = eyes open

**Fig 2:**
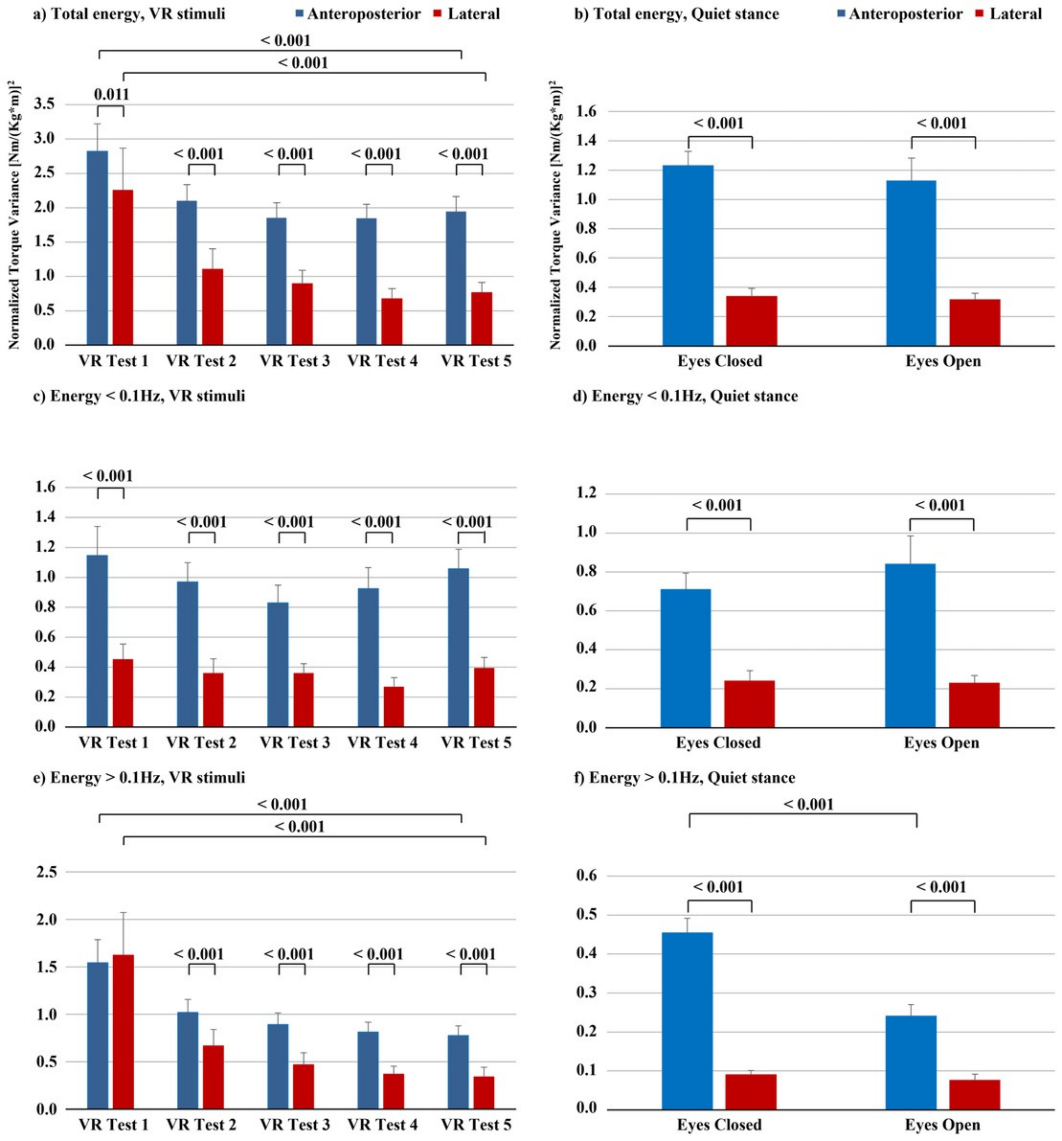
Stability during repeated VR video sessions and during the control tests of quiet stance. The performances recorded during the five repeated VR video sessions are presented as bars (mean) and whiskers (SEM) values of normalized total (A), low frequency (C) and high frequency (E) torque variance. Within the total and high frequency energy spectral ranges, the participants gradually across the sessions significantly reduced the energy used in both anteroposterior and lateral directions. The performance during the quiet stance eyes closed and eyes open control tests are presented as normalized total (B), low frequency (D) and high frequency (F) torque variance. In the control tests, the participants used less high frequency energy with eyes open compared with eyes closed in anteroposterior direction. During almost all VR video sessions and control tests, and in all spectral ranges, less energy was used in lateral direction compared with anteroposterior direction.

Post-hoc analyses performed to determine the effects of adaptation, revealed that the participants significantly reduced the energy used across the five VR video sessions (i.e., energy used during VR video session 1 vs. during VR video session 5) in both anteroposterior and lateral directions. In anteroposterior direction, the total energy was reduced by 31% (p<0.001) and the high frequency energy was reduced by 50% (p<0.001) (Fig 1, Table 3). In lateral direction, the total energy was reduced by 66% (p<0.001) and the high frequency energy was reduced by 79% (p<0.001) (Fig 2, Table 3).

The energy used in VR video session 5 was significantly higher in the total and high frequency energy used in both anteroposterior and lateral directions, compared with the energy used in the control test with eyes open, i.e., where undistorted visual information was available. In anteroposterior direction, the total energy used was 72% higher (p<0.001) and the high frequency energy was 222% higher (p<0.001) during VR video session 5 compared with the eyes open control test (Table 3). In lateral direction, the total energy used was 142% higher (p<0.001) and the high frequency energy was 348% higher (p<0.001) during VR video session 5 compared with eyes open (Table 3).

### Post hoc evaluation of the effects of direction during VR videos

Post-hoc analyses performed to determine the effects of direction on the stability revealed that during VR video session 1, the use of total energy was 20% lower (p = 0.011) in lateral direction than in anteroposterior direction (Fig 1). The use of low frequency energy was 60% lower (p<0.001) in lateral direction than in anteroposterior direction. Across VR video sessions 2–5, the differences in use of energy continued to increase between lateral and anteroposterior directions. In lateral direction the participants used on average 56% less total energy (p<0.001); 63% less low frequency energy (p<0.001), and 48% less high frequency energy (p<0.001).

### Influence of Vision and Direction on quiet stance stability

The repeated measures GLM ANOVA analysis revealed that vision significantly reduced the high frequency energy used with eyes open compared with eyes closed by 31% (p<0.001) (Table 4). The participants used significantly more energy in anteroposterior direction than in lateral direction within all spectral ranges during the control tests; 260% more total energy (p<0.001); 230% more low frequency energy (p<0.001), and 311% more high frequency energy (p<0.001). The interaction between main factors Vision x Direction shows that the high frequency energy used with eyes closed was 405% higher in anteroposterior direction than in lateral direction, and with eyes open 217% higher in anteroposterior direction than in lateral direction (p<0.001).

**Table 4:** Influence of Vision and Direction on quiet stance stability.

Quiet stance stability ^a^	Vision ^b^	Direction ^b^	Vision x Direction ^b^
Total	0.237 [1.5]	**< 0.001 [146.8]**	0.443 [0.6]
Low <0.1 Hz	0.936 [0.0]	**< 0.001 [71.6]**	0.811 [0.1]
High >0.1 Hz	**< 0.001 [37.9]**	**< 0.001 [207.0]**	**< 0.001 [43.5]**

^a^ Repeated measures GLM ANOVA analysis of how the control quiet stance stability was affected by main factors “Vision” and “Direction” alone and by their main factor interactions.

^b^ p-values and [F-values]. The notation “<0.001” means that the p-value is smaller than 0.001. Found significant effects are marked in bold text.

### Post hoc evaluation of the effects of Vision and Direction during quiet stance

Post-hoc analyses revealed that the high frequency energy used was significantly lower with eyes open than with eyes closed in anteroposterior direction by 47% (p<0.001) (Fig 1). The participants used significantly more energy in anteroposterior direction than in lateral direction; 264% more total energy with eyes closed (p<0.001), 257% more total energy with eyes open (p<0.001); 194% more low frequency energy with eyes closed (p<0.001), 265% more low frequency energy with eyes open (p<0.001); 405% more high frequency energy with eyes closed (p<0.001), and 217% more high frequency energy with eyes open (p<0.001).

### Influence of personal characteristics on stability during VR video sessions

Regarding physical status, participants with higher physical activity level, according to Frändin/Grimby questionnaire scores, used less low frequency energy in anteroposterior direction during VR video session 1 (p = 0.033, r_s_ = -0.427). No significant association was seen between the balance confidence, measured with the ABC scale, and the energy used during VR video session 1 (p>0.198) and VR session 5 (p> 0.093), respectively (S1 Table).

The exposure to the VR stimuli made 33% of the participants nauseated during some sessions, but this had no significant association with the stability during VR video session 1 and VR video session 5 (p>0.410) (S1 Table).

## Discussion

This study shows that healthy older adults can adapt fast to distortive visual environments exposed through 3D VR video. This is manifested by a significantly reduced use of energy in both anteroposterior and lateral directions across the five VR video sessions repeated with 10-minute intervals. Thus, the CNS was able to utilize the information provided by the repeated exposures to VR videos into suitable movement strategies that had a simultaneous multidimensionally positive effect on the postural stability in both anteroposterior and lateral directions. However, although the older adults significantly reduced their energy use in both anteroposterior and lateral directions across VR video sessions, the significantly higher percentage of adaptation in lateral direction compared with anteroposterior direction suggests that enhancing the stability in lateral direction might have had priority among the older adults. As both lateral [31, 32] and anteroposterior [33, 34] stability have been identified as risk factors for falls in older adults, it is advantageous that VR offers an approach that addresses multidimensional postural stability deficiencies in older adults. This said, it should be noted that during the initial VR video session, the stability was markedly more affected in lateral direction than in anteroposterior direction (i.e., 612% increase in total energy in lateral direction vs 150% increase in anteroposterior direction compared with the eyes open control test). During VR video session 5, the stability was still significantly affected in both lateral and anteroposterior direction, but the relative difference between directions was smaller (e.g., 142% increase in total energy in lateral direction vs 72% increase in anteroposterior direction compared with the eyes open control test).

When healthy young adults were repeatedly exposed to VR video stimuli during 5 sessions, using a similar protocol, the results obtained were similar to those found in this study, i.e., a fast adaptation caused a reduction in energy use in both anteroposterior and lateral directions [16]. However, the young adults had a higher adaptation in anteroposterior direction compared with lateral direction, and the responses to the VR video stimuli were in general much stronger. One likely reason for this difference might be that the visual environment shown to the young adults influenced them to use another response pattern and adaptation that was perceived as better able to address the immersive visual challenges that they were exposed to. In the present study with older adults, the participants were shown a video of ‘walking in a grocery store”, while in the study with young adults the participants were shown a video of a “roller coaster ride”. From a concept evaluation and protocol design perspective, it would be valuable if there were objective methods that could gauge how strong stability disturbance a certain VR movie produces in anteroposterior and lateral directions. To the best of our knowledge, no such objective measure or method exists to date. However, an important property of an immersive VR movie seems to be whether it produces a perceived self-motion, i.e., “I am moving” and not a perceived environmental motion, i.e., “I am watching camera movements” [35].

The adaptation to the VR video environment affected both the total and high frequency energy used in both directions across VR video session 1 to VR video session 5. An increased use of high frequency energy has a strong causal relationship to being exposed to balance challenges, e.g., when exposed to proprioceptive vibratory stimulation [36] and when affected by physiological issues such as impaired vibration sensation and asymmetrical vestibular function [27, 37]. The activity in the >0.1 Hz frequency range includes both the cognitively produced feedback responses, and the very fast reflexive feedback responses to sensory information where the response processes operate in a faster temporal domain than that can be influenced by causal cognitive processes. The high frequency activity was markedly decreased across the 5 VR video sessions in all statistical tests. However, within the low frequency range the energy levels used across the 5 VR video sessions were significantly decreased only in the GLM ANOVA analysis but not in the post hoc evaluation when comparing the performance during the singled-out VR sessions 1 and 5. This finding contrasts the results obtained when young adults were repeatedly exposed to VR [16]. One reason for this difference might be that the VR movie the young adults watched evoked markedly larger stability challenges, reflected by the 3–4 times larger energy levels reported within all spectral ranges investigated compared with this study. Thus, a significant adaptation aimed at decreasing the low frequency activity might be instigated first when the stability challenges are more prominent. Another reason could be that the low frequency activity is not a direct feedback control response to individual balance perturbations, but instead involves processes used for strategical optimizations, e.g., by altering the postural realignment. This notion is supported by how participants respond to repeated galvanic vestibular and vibratory proprioceptive stimulation [36].

Ageing is associated with degenerative processes which typically affect sensorimotor systems, impair postural stability [9–11] and subsequently increase the risks for falls. Therefore, an adaptation that reduces the high frequency energy during exposure to stability challenges suggests improvements are made in the cognitive and reflexive central processes that handle distorted information [27, 38]. Thus, VR might provide a novel vehicle for rehabilitation, as custom-made visual environments suitable for different kinds of training can be produced artificially, allowing patients to gradually adjust sensorimotor control processes into movement strategies that improve postural stability and prevent falls. Previously, several randomized controlled trials have been performed that used immersive VR as a rehabilitation tool [14, 39–41]. These studies and systematic reviews show improvements in both postural stability (e.g., in posturography assessment and functional balance tests) and in physical performance (e.g., in timed up-and-go test, gait speed, hand grip strength, and anterior reach) for both the VR training group and the control group performing balance exercises or conventional physical therapy [14, 40, 41]. Thus, these studies suggest that, while not being superior to conventional physiotherapy, immersive VR training could serve as a valuable addition to conventional balance rehabilitation protocols.

Vision was found to have a limited effect on postural control during the quiet stance control tests, suggesting that older adults do not always rely on the visual system to serve as the main source of information to maintain postural stability during unchallenging stability conditions. However, when exposed to distortive immersive VR videos the stability was significantly affected, implying that visual information initially was relied heavily upon for controlling the stability, till reevaluation processes changed this view to instead trust other sensory systems and stability maintaining strategies more. Our results are in line with other studies [42, 43], showing that during unchallenging conditions vision may have limited effects on postural sway in older adults. Some studies also suggest that older adults might prioritize information received from the proprioceptive system to maintain postural stability, instead of the visual system [44, 45]. Thus, an impairment in the proprioceptive system, such as a decline in accurate somatosensory information, might result in a disproportionally increased level of postural instability and postural sway in older adults also when visual information is available [27, 38, 43].

The relationship between the stability during VR video session 1 and 5 and the activity level and the balance confidence, respectively, was investigated. Older adults with higher self-reported physical activity levels used less low frequency energy in anteroposterior direction during VR video session 1, indicating that physical activity level seems to be of importance for balance. However, no such relationship was seen between balance confidence and stability during VR video sessions. This could be explained by the high rating of balance confidence in this group, with a mean of 94% (SD 5.7) on the activity-specific balance confidence scale, which indicates that the older adults in this cohort were feeling almost completely safe in their balance confidence. Thus, the relationship between balance confidence and stability during VR could be of importance in a population with lower balance confidence scores and should be further investigated.

During our immersive VR video, a sensory mismatch is produced between the visual illusion of walking around in a grocery store and the inputs from the vestibular and somatosensory systems reporting that the body is not moving. The properties of this VR video exposure and sensory mismatch activated a nausea response [46] in 33% of our participants. However, this nausea had no significant effect on the stability recorded, and thus, we found no causal relationship between being nauseated by the stability challenge and the participant having markedly physical issues with handling the stability challenge. From a concept analysis perspective, it is negative that immersive VR so often causes nausea in older adults. The response of nausea to disruptive visual inflow might signify that CNS has relevant issues with handling certain kinds of visual information, although it does not manifest itself as sensorimotor control deficiency.

### Study limitations

One study limitation is that we did not use the same VR movie as was used when investigating the efficiency of the VR protocol in young adults [16]. The main reason for this is that the VR movie shown to the young adults was so immersive that it, in numerous subjects, caused instability to the level that the operators had to intervene to prevent the participants from falling. Thus, we found it unethical to expose older adults to the same stability challenge and to instead explore in this study whether a VR movie including slower movement illusions still could produce significant postural control reactions detectable by posturography.

Another study limitation is that we only explored the short-term effects of VR video exposure. VR may introduce also long-term improvements that persist over time, for example, manifesting as improved function in daily life, as well as serving a role to prevent falls. We acknowledge the importance of performing long-term studies in the future, but also that these studies should be performed using the most effective protocols. The scope of this study was to explore what characteristics such protocols should encompass, e.g., training intervals, the optimal VR movies to display for different age categories, whether VR may cause secondary effects such as nausea etc.

This study did not include a control group that was not submitted to VR. The study design was instead that two control tests (quiet stance with eyes open and eyes closed) would make each participant serve as their own controls to determine a systematic effect of VR stimuli. However, in this study, the control tests were performed after the VR sessions, which potentially could have affected the results. For future studies, we recommend that the control tests are performed before the VR video sessions or randomly before and after the VR video sessions.

We did not use an extension to the VR headset which would have made it possible to fit the study participants’ own glasses under the VR-headset, this can be seen as a limitation. A study by Huang et al., showed that stability during a static balance test was impaired in the state of myopia [47], thus, we cannot be sure whether not using their own glasses during the test had an impact on their balance. However, despite this we did see significantly reduced amount of energy used for maintaining postural stability from session 1 to 5, indicating that even without glasses they adapted their stability. Recommendations for future studies are to use an extension for glasses to exclude this risk of error.

Another limitation is that we did not screen for cognitive function in the participants. Cognitive impairment could affect balance and increase the risk of falling [48]. Thus, we do not know whether any of our participants had any cognitive impairments. However, all participants were able to get to the lab by themselves and did not need an escort, which could indicate that they had sufficiently good cognitive function. Finally, we investigated if healthy older adults were able to address distortive visual information. These results cannot be generalized to populations suffering from disorders or diseases affecting their postural control. Thus, more studies are needed to establish if multidirectional adaptation to immersive visual VR stimuli occurs in older adults with various pathologies, and to explore future applications of VR in balance training and rehabilitation.

## Conclusion

Repeated exposure to immersive Virtual Reality stimulus can in healthy older adults produce an adaptation that significantly reduces the amount of energy used for maintaining postural stability in an environment with visual distortions. The adaptation detected was multidirectional, immediate, and simultaneous, with a greater reduction of energy used in the lateral direction than in the anteroposterior direction. Vision was found to have a limited effect on postural control during the quiet stance control tests, suggesting that older adults do not always rely on the visual system to serve as the main source of information to maintain postural stability during unchallenging stability conditions. However, when exposed to distortive immersive VR videos the stability was significantly affected, implying that visual information initially was relied heavily upon for controlling the stability, until reevaluation processes changed this view to instead trust other sensory systems and stability maintaining strategies more. Being nauseated during VR videos did not affect stability, but since 33% of the participants in this study became nauseated by the VR video stimuli they were exposed to, nausea might be an effect to be prepared for when older adults are exposed to immersive visual environments.

## Supporting information

S1 Table Association between VR session 1 and 5 and activity level, balance confidence, and nausea during virtual reality exposure. (PDF)
