# Supplementary material for: Video exposure through virtual reality can improve older people’s ability to manage postural instability caused by distortive visual environments
Source: PLoS One. 2024 Aug 21;19(8):e0306834. doi: 10.1371/journal.pone.0306834 (PMC11338449; doi:10.1371/journal.pone.0306834)
Supplement: S1 Table — (PDF) [file pone.0306834.s001.pdf]

	ABC scale[a]		Frändin/Grimby[a]		Nasueated by VR[b]
	r_s	p-value	r_s	p-value	p-value
VR session 1 lateral direction					
Total	-0.143	0.476	-0.111	0.597	0.433
Low <0.1 Hz	-0.103	0.611	-0.197	0.344	0.715
High >0.1 Hz	-0.084	0.677	-0.109	0.603	0.410
VR session 1 anteroposterior direction					
Total	-0.241	0.226	-0.390	0.054	0.781
Low <0.1 Hz	-0.191	0.340	**-0.427**	**0.033**	0.990
High >0.1 Hz	-0.256	0.198	-0.290	0.160	0.623
VR session 5 lateral direction					
Total	-0.081	0.693	-0.251	0.237	0.551
Low <0.1 Hz	-0.106	0.607	-0.241	0.256	0.482
High >0.1 Hz	-0.238	0.242	-0.216	0.310	0.802
VR session 5 anteroposterior direction					
Total	-0.354	0.076	-0.251	0.237	0.958
Low <0.1 Hz	-0.214	0.294	-0.249	0.241	0.499
High >0.1 Hz	-0.336	0.093	-0.365	0.079	0.533

VR=virtual reality, ABC=The Activity-specific Balance Confident scale

[a] *Spearmans rank correlation coefficient*

[b] *Mann-Whitney U test*
